# Event and Comment

**Published:** 1905-12-15

**Authors:** 


### THE
### DENTAL REGISTER.

**Vol. LIX. December 15, 1905. No. 12.**

### EVENT AND COMMENT.

### Tooth=picks Banished.

The Director of the Commons, or student's eating place at the Chicago University, has ordered that no more tooth picks shall be given out in the dining halls. The reasons given are that the use of the tooth pick in public is vulgar, and that the practice is a pernicious habit, because it is unsanitary. Reformers are not living up to their profession unless they are at times spectacular in their actions, and in many cases their zeal is inversely proportionate to their knowledge. Whether the use of the tooth pick in public is vulgar or not depends largely on the manner of its use. It may be used with the utmost decorum as well as benefit, and generally is so used by refined people. The obtrusive person can make as much of a nuisance of a tooth pick as he will of a cigar of a necktie; but should we then say that cigars and neckties must go? Careless people will also mis-apply the tooth pick, especially the cheaply made wooden pick, and do more harm than good by injuring the gums, almost irreparably. There is, however, a most valuable function for a tooth pick, and it should not be bannished from a proper use. It should be of suitable material, a quill for instance, and used with discretion and decency. It is a valuable aid in dislodging impacted food which may injuriously affect the gums, or if left, undergo decomposition, which will result in offensive odors and dangerous chemical reagents. It would be more sensible to instruct Chicago's students in the proper use of the pick than to condemn it entirely.

**Humbuggery.**

The public press prints an item to the effect that a negro applied to a physician in Springfield, Ills., to have a silver plate removed from his head which had been placed under the scalp in order that two horns might be secured to it, to give the appearance of a monstrosity. The crowns of the two upper cuspid teeth had been removed to allow two large tusks to be attached to still further excite the wonder of the credulous public. The negro stated that he had traveled for eighteen months through the South exhibiting himself to large crowds of people as a freak captured in the wilds of Africa. He was compelled to have the plate removed because of the pain he suffered from the irritation. Of course, a moral can be commended to the people who encouraged such deception by their patronage, but as no great harm was done we refrain. But we wonder what the man who was guilty of producing this mutilation of even a willing and avaricious negro could be thinking of, when he could be persuaded to undertake such work! We don't know the name of the dentist or the surgeon who did this work, and we trust it was neither a dentist nor a surgeon. It was a cruel and barbarous operation and whoever did it should be punished. While thinking of this, we find ourselves wondering if some of the operations we find on the teeth are not equally censurable. The gold crowns on exposed teeth, perhaps with diamonds set in them; the hidious crowns and artificial teeth that afford no good function, are essentially humbugs and the dentists who makes them are not good professional men.

**The Year's Work.**

With this issue another volume of the current year's work is completed. It would not be true to say that we have made a complete record of all the progress of the year. Under our limitations such would be impracticable, and we very much doubt if any editor feels that he has placed before his readers all the important events of the year. As we review the work of the year we think the profession should be pleased at the substantial advancement made in all departments of practice. The discussion of porcelain as a filling material, which engrossed the profession largely last year has perhaps not occupied so prominent a place this year, but it has caused a closer study of all other filling materials, as they have been placed on the defensive. Gold, amalgam, and cement have all been given unusual attention. Gold fillings have been made as clinics before the dental societies all over the country in greater numbers than for many years. Amalgam and cements have been studied scientifically as never before, and there seems to be a promise that these materials will be better understood and manufactured in the near future. Orthodontia and prophylaxis continue to command attention, and the results of the earnest work being done on these two subjects will be to materially elevate the technical standards in both, and reflexly, to stimulate greater devotion to move thorough efforts to save all the natural teeth in their perfect form and natural relations.

Perhaps the most important event of the year has been the advancement made in educational standards. Many thought the colleges made a retrograde step last year in returning to a three-year course of required study. It is now the general belief that a substantial advance has been made, since more than half of the colleges have lengthened their terms to eight months, and beginning with the next entering class, practically a high school education will be required for admission. This will place a greater burden on the schools, as it means more expense for instruction, and will probably reduce somewhat the number of students entering college. Several new laws have been enacted during the year, and now practically every state requires that graduates shall pass examinations before the state board before registering for practice. The result of this will be to stimulate colleges to turn out men capable of passing these examinations, as no school can afford to have its graduates discredited in any considerable number. This educational advance of course can not now be adequately estimated, but it seems logical that we should make a great gain under these circumstances. There can scarcely be a question as to the ultimate successful issue of this movement. It was thought that the International Congress of last year would exhaust the literary output for a few years, but such does not seem to have been the case. The Lewis & Clark Dental Congress, the National, District and State meetings, and the journals have all been well sustained, with well prepared and interesting papers and discussions. Altogether the year has been a very satisfactory one.

